# Patient-evaluated cognitive function measured with smartphones and the association with objective cognitive function, perceived stress, quality of life and function capacity in patients with bipolar disorder

**DOI:** 10.1186/s40345-020-00205-1

**Published:** 2020-10-30

**Authors:** Maria Faurholt-Jepsen, Kamilla Woznica Miskowiak, Mads Frost, Ellen Margrethe Christensen, Helga Þórarinsdóttir, Jakob Eyvind Bardram, Maj Vinberg, Lars Vedel Kessing

**Affiliations:** 1 Copenhagen Affective Disorder Research Center (CADIC), Psychiatric Center Copenhagen, Rigshospitalet, Blegdamsvej 9, 2100 Copenhagen, Denmark; 2 Monsenso Aps, Langelinie Alle 47, Copenhagen, Denmark; 3 Department of Health Technology, Technical University of Denmark, Lyngby, Denmark

**Keywords:** Bipolar disorder, Cognitive function, Smartphone

## Abstract

**Background:**

Cognitive impairments in patients with bipolar disorder (BD) have been associated with reduced functioning. Aims: To investigate the association between (1) patient-evaluated cognitive function measured daily using smartphones and stress, quality of life and functioning, respectively, and (2) patient-evaluated cognitive function and objectively measured cognitive function with neuropsychological tests.

**Methods:**

Data from two randomized controlled trials were combined. Patients with BD (N = 117) and healthy controls (HC) (N = 40) evaluated their cognitive function daily for six to nine months using a smartphone. Patients completed the objective cognition screening tool, the Screen for Cognitive Impairment in Psychiatry and were rated with the Functional Assessment Short Test. Raters were blinded to smartphone data. Participants completed the Perceived Stress Scale and the WHO Quality of Life questionnaires. Data was collected at multiple time points per participant. p-values below 0.0023 were considered statistically significant.

**Results:**

Patient-evaluated cognitive function was statistically significant associated with perceived stress, quality of life and functioning, respectively (all p-values < 0.0001). There was no association between patient-evaluated cognitive function and objectively measured cognitive function (*B:0.0009, 95% CI 0.0017; 0.016, p *= *0.015*). Patients exhibited cognitive impairments in subjectively evaluated cognitive function in comparison with HC despite being in full or partly remission (*B:* − *0.36, 95% CI* − *0.039;* − *0.032, p *< *0.0001*).

**Conclusion:**

The present association between patient-evaluated cognitive function on smartphones and perceived stress, quality of life and functional capacity suggests that smartphones can provide a valid tool to assess disability in remitted BD. Smartphone-based ratings of cognition could not provide insights into objective cognitive function.

## Background

Bipolar disorder (BD) is estimated as one of the most important causes of disability worldwide (Pini et al. 2005; Vos et al. 2012). BD is characterized by recurrent episodes of depression, (hypo)mania and mixed episodes intervened by periods of euthymia (Goodwin and Jamison 1996) and with a high degree of comorbidity and functional impairment (Sanchez-Moreno et al. 2009). In addition to the affective symptoms, patient with BD often experience cognitive deficits (Martinez-Aran et al. 2000, 2004) even during periods of remission (Kessing 1998; Torres et al. 2007; Bourne et al. 2013; Jensen et al. 2016). Cognitive dysfunction in patients with BD is associated with prolonged illness duration, reduced chances of recovery independently of affective symptoms (Etkin et al. 2013), and impaired quality of life and social functioning (Jensen et al. 2016; Dion et al. 1988; Dickerson et al. 2001; Mackala et al. 2014). Recent studies have indicated that cognitive deficits in patients with BD are among the strongest predictors of functional impairments (Martínez-Arán et al. 2004; Baune and Malhi 2015). Thus, correct identification and treatment of cognitive dysfunction is of major clinical importance.

The relationship between objectively assessed cognitive function with neuropsychological tests and patient-evaluated cognitive function is debatable with studies presenting disparity in findings. The majority of studies have found an absence of a relationship between these measures (Demant et al. 2015; Martinez-Aran et al. 2005; Svendsen et al. 2012; van der Werf-Eldering et al. 2011; Burdick et al. 2005), while one has found a significant association (Arts et al. 2011). Overall, it therefore seems that patients with the most subjective cognitive complaints may not always display the greatest objective cognitive impairments and vice versa. This is major challenge in the clinical assessment of cognitive functions in patients with BD (Miskowiak et al. 2018). New tools to get insights into patients’ cognitive function in daily life and how it relates to quality of life and functional capacity would thus improve identification of cognitive impairments in the clinic and inform clinicians about their clinical importance (Miskowiak et al. 2018).

Ecological momentary assessments (EMA) reflect the methods used to collect assessments of individuals’ real-time states repeatedly over time during naturalistic settings, and may reduce recall bias (Shiffman et al. 2008). Today a median of 76% of adults across 18 advanced economies reported having a smartphone (Taylor and Silver 2019), and many people use a smartphone on a daily basis (Ericsson Mobility Report November. 2018). The rapid evolution and ubiquity of mobile networks have resulted in increasing growth of e-mental health technologies, including electronic platforms offering tolls for remote self-monitoring (Lal and Adair 2014). By using daily smartphone-based self-monitoring potential recall bias in self-reported patient data is minimized. Thus, smartphones extends the use of EMA beyond its classical use for self-reports and offer the opportunity to collect fine-grained data unobtrusively and outside the clinical settings (Ebner-Priemer and Trull 2009).

However, no prior study has collected data on patient-evaluated daily smartphone-based measures of cognitive function. The present study therefore explores how patients’ smartphone-based evaluations of cognitive function related to perceived stress, quality of life, and functional capacity as well as its association with objectively measured cognitive function on neuropsychological tests. Data in the present study were collected during two randomized controlled trials (RCT) investigating the effect of daily smartphone-based self-monitoring in patients with BD (Faurholt-Jepsen et al. 2014, 2019).

### Aims and hypotheses

The present study aimed to (1) investigate the associations between daily patient-evaluated cognitive function measured using smartphones and perceived stress, quality of life and functioning, respectively, in patients with BD, (2) examine the association between patient-evaluated daily cognitive function measured using smartphones and objectively-measured cognitive function in patients with BD, and (3) examine whether group status (patient versus healthy control individuals (HC)) interacted with the associations between perceived stress, quality of life and functioning. The analyses were based on patient-reported data on cognitive function measured daily using smartphones and objectively-measured cognitive function, clinically rated depressive and manic symptoms and functioning as well as patient-based questionnaires concerning perceived stress and quality of life collected at multiple time points for each patient during follow-up.

Based on prior evidence we hypothesized that (1) impaired daily patient-evaluated cognitive function would be associated with increased perceived stress, and with decreased quality of life and functional capacity in patients with BD, (2) there would be no association between patient-evaluated daily cognitive function measured using smartphones and objectively-measured cognitive function, and (3) patients would report poorer cognitive function compared with HC, and the associations between daily evaluated cognitive function and perceived stress, quality of life and functioning, respectively, in patients with BD and HC would be different.

## Methods

### Participants, settings and design

The present study combines data collected as part of two randomized controlled trials (RCTs) investigating the effect of smartphone-based monitoring in patients with BD (the MONARCA I trial and the MONARCA II trial) (Faurholt-Jepsen et al. 2014, 2019). In addition, a group of HC collecting smartphone-based data on cognitive function was included for comparison (Kessing et al. 2017). Thus, data was collected as part of studies published in study protocols á priori.

### Patients with bipolar disorder

The MONARCA I trial: The patients were recruited from The Copenhagen Clinic for Affective Disorders, Copenhagen, Denmark during a period from September 2011 to March 2013. The clinic is a specialized outpatient clinic with a catchment area consisting of the Capital Region in Denmark corresponding to 1.4 million people. Patients with a newly diagnosis of BD or with treatment-resistant BD were referred to the clinic. The staff consists of specialists in psychiatry, psychologists, nurses, and a social worker, all with specific experience and knowledge regarding BD. Treatment at the clinic comprises a 2 years program including combined evidence-based psychopharmacological treatment and supporting therapy, including group psychoeducation (Kessing et al. 2013). The trial had a six-month follow-up period. Inclusion criteria: BD diagnosis according to ICD-10 using the Schedules for Clinical Assessment in Neuropsychiatry (SCAN) interview (Wing et al. 1990), age between 18 and 60 years, a Hamilton Depression Rating Scale 17-item (HDRS-17) score ≤ 17 (Hamilton 1967) and Young Mania Rating Scale (YMRS) score ≤ 17 (Young et al. 1978) at the time of inclusion. Exclusion criteria: Pregnancy, a lack of Danish language skills, inability to learn the technicalities for using a smartphone, unwilling to use the trial smartphone as the primary cell phone, and severely physical illness or schizophrenia, schizotypal or delusional disorders according to the SCAN interview.

The MONARCA II trial: All patients with a diagnosis of BD who had previously been treated at the Copenhagen Clinic for Affective Disorder, Copenhagen, Denmark (as described above) in the period from 2004 to January 2016 and who at the time of recruitment were being treated at community psychiatric centres, private psychiatrists and general practitioners were invited to participate in the trial. Patients were included in the study for a nine-month follow-up period if they had a BD diagnosis according to ICD-10 using the Schedules for Clinical Assessments in Neuropsychiatry (SCAN) (Wing et al. 1990) and previously were treated at the Copenhagen Clinic for Affective Disorder. Patients with schizophrenia, schizotypal or delusional disorders, previous use of the MONARCA system, pregnancy and lack of Danish language skills were excluded. Patients with other comorbid psychiatric disorders and substance use were eligible for the trial.

As part of the MONARCA I trial and the MONARCA II trial, patients were randomized to either using a smartphone-based monitoring system (the Monsenso system) for daily self-monitoring (the intervention group) or to treatment as usual (the control group). Patients included in the intervention group from both trials collected daily smartphone-based self-monitoring data on cognitive function and were included in the analyses in the present report. Inclusion and exclusion criteria were investigated and assessed by two clinical researchers (MFJ and ASJ).

### Healthy control individuals

In the present study, a group of HC was included in the analyses to investigate differences in the association between cognitive functioning and stress, quality of life and functioning, respectively with those in the patient population. The HC were part of a larger cohort study (Kessing et al. 2017) and recruited consecutively from the Blood Bank at Rigshospitalet, Copenhagen University Hospital, Denmark, by approaching blood donors in the waiting room on random occasions from September 2015 to August 2016. The inclusion criteria were: age over 18 years, no history of psychiatric illness and no first-generation family history of psychiatric illness. The exclusion criteria were: lack of Danish language skills and pregnancy. The healthy individuals participated as part of a larger cohort study (Kessing et al. 2017).

### Daily smartphone-based monitoring

On a daily basis during the follow-up, participants in the trials used a smartphone with the Monsenso app installed and were instructed to use the system for evaluation (Faurholt-Jepsen et al. 2014). The app allowed for daily evaluation of cognitive function (evaluated from not present, present to some degree or present (scale: 0, + 1, + 2)). Further details regarding the Monsenso system are described elsewhere (Faurholt-Jepsen et al. 2014).

### Clinical measurements

In the MONARCA I trial outcome measurements were conducted monthly for the entire trial period of 6 months. In the MONARCA II trial outcome measurements were conducted at baseline, after 4 weeks, 3 months, 6 months and 9 months. The HC used the smartphone-based system on a daily basis for 4 months and outcome measurements were conducted at baseline and after 4 months.

All clinical assessments were conducted by researchers (MFJ and HÞ), who were blinded to all smartphone-based data. Thus, data on severity of depressive and manic symptoms as well as functioning were collected rater-blinded.

Clinical rater-blinded assessments: The severity of depressive and manic symptoms were clinically assessed using the HDRS (Hamilton 1967) and the YMRS (Young et al. 1978). Functioning were clinically rated using the Psychosocial Assessment Short Test (FAST), which is an 24-item interviewer-administrated interview concerning autonomy, occupational functioning, cognitive functioning, financial issues, interpersonal relationships and leisure time (Rosa et al. 2007). Each item is scored from 0 to 3. Higher scores indicate functional impairment and scores above 11 indicate impaired functioning.

Patient-reported questionnaires: The following questionnaires were filled in by the patients at all visits with the researcher: Perceived stress according to Cohen’s Perceived stress scale (PSS), which is a 14-item questionnaire measuring the degree to which situations in one’s life are appraised as stressful. Each item is scored from 0 to 4. Higher scores indicate higher perceived stress (Cohen et al. 1983); Quality of life according to WHO Quality of Life-BREF (WHOQoL), which is a 26-item questionnaire concerning physical health, psychological health, social relationships, environment. Each item is scored from 1 to 5. Higher score indicates better quality of life (WHO 1998).

Assessments of cognitive function: As part of the MONARCA I trial objectively-evaluated cognitive function with the Screen for Cognitive Impairment in Psychiatry (SCIP) (Guilera et al. 2009; Rojo et al. 2010) and patient-evaluated cognitive function with the Massachusetts General Hospital Cognitive and Physical Functioning questionnaire (CPFQ) (Fava et al. 2009) were collected at baseline, after 3 months and 6 months. The SCIP consist of five short performance-based tests that assesses verbal learning, delayed verbal memory, working memory, verbal fluency and processing speed with a high validity and reliability in patients with BD (Guilera et al. 2009; Jensen et al. 2015). The SCIP exists in three parallel versions to minimize learning effects with repeated testing, and in the present study the administration order of the SCIP versions was defined using a randomization list to randomly one of the three SCIP versions to each patient during follow-up. The CPFQ is a 7-item questionnaire which measures patients’ experience of cognitive and physical symptoms. The CPFQ was included to increase the internal validity of the findings by investigating a well-known subjective measure of cognition against both the smartphone-based cognitive function as well as the performance-based cognition measure, SCIP.

An overview of assessments during the studies is presented in Table 1.

**Table 1:** Assessments during the MONARCA studies

	The MONARCA I trial	The MONARCA II trial
Smartphone-based daily ratings	X	X
The hamilton depression rating scale	Monthly	0, 1, 3, 6 and 9 months
The young mania rating scale	Monthly	0, 1, 3, 6 and 9 months
The functional assessment short test	Monthly	0, 1, 3, 6 and 9 months
The perceived stress scale	Monthly	0, 1, 3, 6 and 9 months
Screen for cognitive impairment in psychiatry	0, 3 and 6 months	–
The Massachusetts general hospital cognitive and physical functioning questionnaire	0, 3 and 6 months	–

### Statistical methods

The hypotheses and statistical analyses for the present study were defined á priori.

Since the PSS questionnaire reflects perceived stress during the two previous weeks, the WHOQoL questionnaire reflects quality of life for the previous 2 weeks, and the FAST rating scale reflects psychosocial functioning during the previous week, mean measures of cognitive function measured using smartphones for the days the scales were reflecting were used in the present report. Calculated total scores for the PSS and the WHOQoL were used in the present study. Since five subitems on the FAST scale reflect cognitive function, in addition to total scores of the FAST, analyses excluding measures on cognitive function (item 10, 11, 12, 13 and 14) on the FAST scale as well as analyses only including measures of cognitive function (item 10, 11, 12, 13 and 14) were conducted in the present study.

In relation to aim 1: To investigate the association between both objectively-measured cognitive function and PSS, WHOQoL and FAST, respectively as well as the association between patient-evaluated cognitive function and PSS, WHOQoL and FAST, respectively, a two-level linear mixed effect model was employed (Tables 2 and 3). In all models, we first considered an unadjusted model (model 1). Secondly, we considered a model adjusted for age and gender as possible covariates. Thirdly, we considered a model adjusted for age, gender, and severity of depressive and manic symptoms according to HDRS and YMRS, respectively as possible covariates. In sensitivity analysis, we restricted all statistical analyses in relation to aim 2 (Tables 2 and 3) to include patients with a HDRS score ≤ 14 and a YMRS score ≤ 14, and in addition for the results presented in Table 4 to include patients with a HDRS score ≤ 7 and a YMRS score ≤ 7.

**Table 2:** Associations between objective clinically evaluated cognitive function and patient evaluated perceived stress, quality of life and functioning, respectively in patients with bipolar disorder, N = 67

	Model 1^a^			Model 2^a^		
	B	95% CI	p	B	95% CI	p
SCIP^b^						
PSS	− 0.23	− 0.45; − 0.0084	0.042	− 0.22	− 0.43; − 0.013	0.038
WHOQoL	0.15	− 0.0043; 0.30	0.057	0.14	− 0.010; 0.28	0.067
FAST	− 0.13	− 0.28; 0.013	0.075	− 0.13	− 0.28; 0.011	0.072
HDRS	− 0.28	− 0.55; − 0.0072	0.044	− 0.27	− 0.54; − 0.0024	0.048
YMRS	− 0.11	− 0.47; 0.25	0.55	− 0.15	− 0.50; 0.20	0.40

^a^ Model 1: Unadjusted. Model 2: Adjusted for age and gender. SCIP: The Screen for Cognitive Impairment in Psychiatry; PSS: Perceived stress measured using Cohen’s Perceived Stress Scale; WHOQoL: Quality of life measures using WHO Quality of Life BREF; FAST: Psychosocial functioning measured using Functional Assessment Short Test; HDRS: Hamilton Depression Rating Scale; YMRS: Young Mania Rating Scale

**Table 3:** Associations between patient-evaluated cognitive function measured using smartphone and patient-evaluated perceived stress, quality of life and functioning, respectively in patients with bipolar disorder, N = 117

	Model 1^a^			Model 2^a^			Model 3^a^		
	B	95% CI	p	B	95% CI	p	B	95% CI	p
Cognitive function measures using smartphones^b^									
PSS	0.033	0.024; 0.042	< 0.0001	0.033	0.024; 0.042	< 0.0001	0.027	0.017; 0.036	< 0.0001
WHOQoL	− 0.023	− 0.031; − 0.016	< 0.0001	− 0.023	− 0.031; − 0.016	< 0.0001	− 0.024	− 0.032; − 0.017	< 0.0001
FAST	0.021	0.014; 0.027	< 0.0001	0.022	0.015; 0.028	< 0.0001	0.014	0.0062; 0.021	< 0.0001
FAST excluding cognitive items (item 10–14)	0.023	0.015; 0.031	< 0.0001	0.023	0.015; 0.032	< 0.0001	0.017	0.0087; 0.025	< 0.0001
FAST including cognitive items (item 10–14)	0.066	0.044; 0.088	< 0.0001	0.069	0.047; 0.091	< 0.0001	0.041	0.018; 0.064	0.001

^a^Model 1: Unadjusted. Model 2: Adjusted for age and gender. Model 3: Adjusted for age, gender, Hamilton Depression Rating Scale 17-items score and Young Mania Rating Scale score. ^b^Scored on a scale from 0, 1, 2. PSS: Perceived stress measured using Cohen’s Perceived Stress Scale; WHOQoL: Quality of life measures using WHO Quality of Life BREF; FAST: Psychosocial functioning measured using Functional Assessment Short Test

**Table 4:** Associations between patients-evaluated cognitive function and objective clinically evaluated cognitive function in patients with bipolar disorder

	Model 1^a^			Model 2^a^			Model 3^a^		
	B	95% CI	p	B	95% CI	p	B	95% CI	p
Cognitive function measured using smartphones^b^, n = 33									
SCIP, overall score^c^	− 0.00075	− 0.0096; 0.0081	0.87	0.0017	− 0.0078; 0.011	0.73	0.00090	0.0017; 0.016	0.015
Verbal learning and memory	0.0041	− 0.022; 0.030	0.76	0.0080	− 0.018; 0.034	0.55	0.025	0.0050; 0.045	0.013
Delayed memory	− 0.0063	− 0.039; 0.026	0.71	0.0038	− 0.031; 0.039	0.83	0.033	0.0055; 0.061	0.018
Working memory	0.0032	− 0.016; 0.023	0.75	0.0054	− 0.014; 0.025	0.60	0.0031	− 0.14; 0.021	0.72
Word mobilization	− 0.037	− 0.051; 0.044	0.88	0.0031	− 0.045; 0.052	0.90	0.031	− 0.0067; 0.069	0.11
Processing speed	− 0.032	− 0.074; 0.011	0.14	− 0.026	− 0.076; 0.023	0.30	− 0.0068	− 0.051; 0.037	0.76
CPFQ^d^	0.023	0.014; 0.032	< 0.0001	0.023	0.014; 0.032	< 0.0001	0.019	0.0087; 0.030	< 0.0001
CPFQ^d^ n = 67									
SCIP, overall score^c^	− 0.091	− 0.20; 0.022	0.11	− 0.077	− 0.20; 0.042	0.21	− 0.00030	− 0.092; 0.092	0.99
Verbal learning and memory	− 0.49	− 0.82; − 0.15	0.005	− 0.46	− 0.82; − 0.11	0.010	− 0.018	− 0.30; 0.27	0.90
Delayed memory	0.014	− 0.30; 0.39	0.94	0.069	− 0.33; 0.46	0.73	0.13	− 0.17; 0.43	0.40
Working memory	0.016	− 0.21; 0.24	0.89	0.023	− 0.21; 0.26	0.85	0.0065	− 0.20; 0.18	0.95
Word mobilization	− 0.30	− 0.31; − 0.09	0.024	− 0.67	− 1.31; − 0.26	0.041	− 0.087	− 0.59; 0.42	0.74
Processing speed	− 0.50	− 1.06; 0.051	0.075	− 0.43	− 1.06; 0.20	0.18	− 0.19	− 0.66; 0.29	0.44

^a^ Model 1: Unadjusted. Model 2: Adjusted for age and gender. Model 3: Adjusted for age, gender, Hamilton Depression Rating Scale 17-item score and Young Mania Rating Scale score ^b^ Scored on a scale from 0, 1,2 ^c^ SCIP: The Screen for Cognitive Impairment in Psychiatry ^d^ CPFQ: The Massachusetts General Hospital Cognitive and Physical Functioning Questionnaire

In relation to aim 2: To investigate the association between patient-evaluated cognitive function and objectively-measured cognitive function a two-level linear mixed effect model, which accommodates both variation of the variables of interest within patients (intra-individual variation) and between individuals (inter-individual variation) was employed. The models included a fixed effect of time (measurement visit number) and a patients-specific random effect allowing for individual intercept and a slope for each participant (Table 4). In addition, the association between these measures according to the sub-domains on the SCIP reflecting verbal learning and memory, delayed memory, working memory, verbal fluency and processing speed were employed. Model 1 reflects unadjusted analyses, model 2 includes age and gender as covariates, and model 3 includes age, gender, HDRS and YMRS score as covariates.

In relation to aim 3: To investigate differences in the association between cognitive function measures using smartphones and perceived stress, quality of life and functioning in patients with BD and HC, interactions between the groups (BD or HC) and outcome measures were investigated for each of the considered models and reported accordingly.

As few prior studies have investigated associations between daily patient-reported cognitive function and perceived stress, quality of life and functioning, respectively, in patients with BD, we were not able to make statistical power analyses prior to the study since potential effects were unknown. However, to account for multiple testing in the statistical models the Bonferroni correction method was used. Thus, p-values below 0.0023 in the individual analyses were considered statistically significant. Model assumptions were checked visually by means of residuals and QQ plots for each of the statistical analyses. Data were entered using Excel and Epidata^®^, STATA (StataCorp LP, College Station, TX, USA) version 13 was used for statistical analyses.

### Ethical considerations

The trials were approved by the Regional Ethics Committee in the Capital Region of Denmark (H-2-2011-056, H-2-2014-059 and H-7-2014-007) and the Danish Data protection agency (2013-41-1710). The law on handling of personal data was respected. Prior to commencement the trials were registered at ClinicalTrials.gov (NCT01446406 and NCT02221336). Electronic data collected from the smartphones were stored at a secure server at Concern IT, Capital Region, Denmark (I-suite number RHP-292 2011-03). The trial complied with the Helsinki Declaration of 1975, as revised in 2008.

## Results

### Background characteristics

In the MONARCA I trial, a total of 123 potential participants with BD receiving treatment at the Copenhagen Clinic for Affective Disorder, Denmark at the time of the study were assessed for eligibility. Among these, 78 patients (63.4%) were included. A total of 33 patients in the intervention group of the MONARCA I trial provided patient-evaluated cognitive function measured daily using smartphone-based data which was included in the present study.

**Fig. 1:**
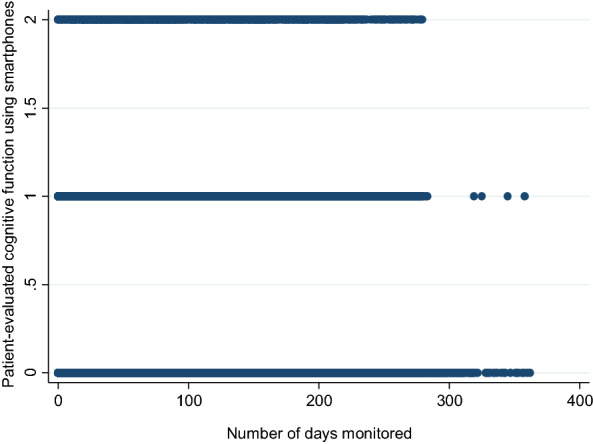
Patient-evaluated cognitive function measured daily using the Monsenso smartphone-based system, N = 117

In the MONARCA II trial, a total of 735 patients with BD previously receiving treatment at the Copenhagen Clinic for Affective Disorder, Denmark were assessed for eligibility. Of these 544 patients were not included due to; unable to get in contact with the patient (n = 240), declined to participate (main reasons: did not have the time, did not want to participate in a research study or had moved too far away making transportation a problem) (n = 282 patients), or were excluded due to previous use of the MONARCA system (n = 22). A total of 84 patients in the intervention group of the MONARCA II trial provided smartphone-based data on level of patient-evaluated cognitive function measured daily using the Monsenso smartphone-based system and was included in the present study. Figure 1 present level of patient-evaluated cognitive function measured using smartphones in the included patient population (combined populations from the MONARCA I trial and the MONARCA II trial) (N = 117).

A total of 255 HC blood donors were approached at random occasions at the Blood bank at Rigshospitalet, Copenhagen during a 9-month recruitment period. Over half of the HC (n = 129) were ineligible to participate and another 49 were not interested in participating in the study or did not have the time to participate. Of the remaining 77 individuals, 46 were included in the study. Six of those were not included in the final cohort due to a lack of smartphone data. Thus, the final cohort of HC comprised 40 HC.

As seen from Table 5, patients had a mean age of 30.9 (SD 9.9) years (HC: 35.2 (SD 12.8) years) and 62.4% (n = 73) were women (HC: 55% (n = 22)). During the study the patients had a median HDRS score of 7 [IQR 3-14] and a median YMRS score of 2 [IQR 0-4]. There were statistically significant differences in background characteristics between patients with BD and HC. At baseline the patients had a FAST score of 21.0 (SD 14.7) (HC: 1.61 (SD 2.10)) corresponding to moderate impaired functional capacity using a standardized cut-off defined by others (Bonnín et al. 2018). There was an adherence to patient-evaluated cognitive function of 66.4% of the days.

**Table 5:** Background characteristics of patients with bipolar disorder (BD) and healthy control individuals (HC) using smartphones for daily self-monitoring, N = 157

	BD, The MONARCA I trial, n = 33	BD, The MONARCA I & II trials, n = 117	HC, n = 40	p-value^*^
Age, years	29.1 (7.5)	30.9 (9.9)	35.2 (12.8)	0.028
Female gender, % (n)	65.7 (22)	73 (62.4)	55 (22)	0.0063
Full time employed, % (n)	14.3 (5)	17.1 (20)	57.5 (23)	< 0.0001
PSS score^a^	18.32 (9.22)	16.30 (8.81)	6.87 (5.04)	< 0.0001
CPFQ score^b^	21.36 (6.74)	–	–	–
WHOQoL score^c^	81.58 (13.41)	86.76 (13.01)	97.79 (7.37)	< 0.0001
FAST score^d^	23.22 (16.65)	21.04 (14.68)	1.61 (2.10)	< 0.0001
SCIP score^e^	73.80 (11.32)	–	–	–
HDRS, follow-up^f^	9.23 (7.27)	8.77 (7.13)	0.25 (0.54)	< 0.0001
YMRS, follow-up^g^	3.14 (4.37)	3.07 (4.32)	0.071 (0.27)	< 0.0001

Data are mean (SD), median [IQR] or proportions (n) unless otherwise stated

^a^PSS: Perceived stress measured using Cohen’s Perceived Stress Scale; ^b^CPFQ: The Massachusetts General Hospital Cognitive and Physical Functioning Questionnaire; ^c^WHOQoL: Quality of life measures using the WHO Quality of Life BREF; ^d^FAST: Psychosocial functioning measured using the Functional Assessment Short Test; ^e^SCIP: The Screen for Cognitive Impairment in Psychiatry; ^f^HDRS: Hamilton Depression Rating Scale 17-items; ^g^YMRS: Young Mania Rating Scale

^*^Difference between patients with BD (The MONARCA I & II trials) versus the HC

### Patient-evaluated cognitive function and perceived stress, quality of life and functioning

In both the unadjusted models and the models adjusted for age, gender and scores on the HDRS and YMRS, there was a statistically significant positive association between patient-evaluated cognitive function measured using smartphones and scores on the PSS (fully adjusted model: *B:0.027, 95% CI 0.017; 0.036, p *< *0.0001*). Thus, for every increase of 10 point on the PSS scale, there was an increase of 0.17 on scores of patient-evaluated cognitive function measured smartphones (scale: 0 to 2). Further, in both the unadjusted and the models adjusted for age, gender and scores on the HDRS and YMRS, there was a statistically significant negative association between patient-evaluated cognitive function measured using smartphones and scores on the WHOQoL (fully adjusted model: *B:− 0.024, 95% CI − 0.032; − 0.017, p *< *0.0001*). In addition, in both the unadjusted and the models adjusted for age, gender and scores on the HDRS and YMRS, there was a statistically significant positive association between patient-evaluated cognitive function measured using smartphones and scores on the FAST (fully adjusted model: *B:0.014, 95% CI 0.0062; 0.021, p *< *0.0001*)–including in analyses using FAST scores excluding the cognitive items (item 10-14) (p < 0.0001) and in analyses only including the cognitive item (p = 0.001) (Table 3).

In sensitivity analyses, restricting all of the statistical analyses listed above to only include patients with a HDRS score ≤ 14 and a YMRS score ≤ 14 did not alter the estimates to a large extent (PSS, adjusted model: *B:0.027, 95% CI 0.017; 0.037, p *< *0.0001*; WHOQoL, adjusted model: *B:− 0.020, 95% CI − 0.028; − 0.012, p *< *0.0001;* FAST, adjusted model: *B:0.017, 95% CI: 0.010; 0.023, p *< *0.0001;* FAST without cognitive items, adjusted model: *B:0.018, 95% CI: 0.010; 0.026, p *< *0.001*). In further sensitivity analyses, restricting all of the statistical analyses listed above to only include patients with a HDRS score ≤ 7 and a YMRS score ≤ 7 did not alter the estimates to a large extent (PSS, adjusted model: *B:0.027, 95% CI: 0.018; 0.036, p *< *0.0001*; WHOQoL, adjusted model: *B:− 0.030, 95% CI − 0.037; − 0.022, p *< *0.0001;* FAST, adjusted model: *B:0.0084, 95% CI 0.00035; 0.017, p *= *0.041*).

### Patient-evaluated cognitive function versus objectively measured cognitive function

There was no statistically significant association between patient-evaluated cognitive function measured using smartphones and objectively-measured cognitive function using the SCIP in unadjusted analyses and in analyses adjusted for age and gender (and in models adjusted for age, gender and scores on the HDRS and the YMRS) (model 2: *B: 0.0017, 95% CI − 0.0078; 0.011, p *= *0.73*) (Table 4). In addition, there was no statistically significant association between patient-evaluated cognitive function measured using the CPFQ and objectively measured cognitive function using the SCIP (*p *= *0.21*). There were no statistically significant associations between sub-domains on the SCIP and patient-evaluated cognitive function measured using smartphones and the CPFQ, respectively (Table 4). There was a statistically significant association between patient-evaluated cognitive function measured using smartphones and patient-evaluated cognitive function measured using the CPFQ (model 3: *B: 0.019, 95% CI 0.0087; 0.030, p *< *0.0001*).

There were no statistically significant associations between objectively-measured cognitive function using the SCIP and PSS, WHOQoL and FAST–including analyses using FAST without the cognitive items (all p-values > 0.03) (Table 4). In sensitivity analyses, restricting the statistical analyses to include patients with a HDRS score ≤ 14 and a YMRS score ≤ 14 there were no statistically significant associations between objectively measured cognitive function using the SCIP and PSS, WHOQoL and FAST. PSS, adjusted model: *B:− 0.13, 95% CI − 0.38; 0.12, p *= *0.31*; WHOQoL, adjusted model: *B:0.054, 95% CI − 0.11; 0.22, p *= *0.53;* FAST, adjusted model: *B:− 0.022, 95% CI − 0.024; 0.17, p *= *0.80*.

In addition, there were no statistically significant associations between objective clinically evaluated cognitive function and HDRS and YMRS, respectively (all *p* value > 0.04) (Table 4).

### Patients with bipolar disorder versus healthy control individuals

Patients with BD reported a statistically significant lover mean level of cognitive function measured using smartphones compared with the HC (*B: − 0.36, 95% CI − 0.039; − 0.032, p *< *0.0001*).

When investigating the association between cognitive function measured using smartphones and PSS, WHOQoL and FAST, there was a statistically significant interaction between between the groups (BD or HC) and the outcome measures. Thus, results are reported separately (for the association between cognitive function measured using smartphones and PSS, WHOQoL and FAST in patients with BD, see results section above).

In HC, in both the unadjusted and the models adjusted for age and gender, there was no statistically significantly associations between cognitive function measured smartphones and scores on the PSS (*p *= *0.61*), the WHOQoL (*p *= *0.75*), and the FAST (*p *= *0.55*).

## Discussion

This is the first study to investigate associations between fine-grained real-time collected data on daily patient-evaluated cognitive function measured with smartphones and objectively-measured cognitive function, perceived stress, quality of life and functional capacity in patients with BD. Notably, the majority of patients included in the present study were in full or partly in remission. Interestingly and as hypothesized, we found that impaired daily patient-evaluated cognitive function was associated with increased perceived stress, decreased quality of life and functioning in patients with BD. In contrast, daily patient-evaluated cognitive function measured using smartphones was not associated with objectively measured cognitive function measured using the SCIP. Similarly, there was no association between patient-evaluated cognitive function measured using questionnaires (CPFQ) and objectively measured cognitive function using the SCIP, but there was an association between patient-evaluated cognitive function measured using smartphones and patient-evaluated cognitive function measured using questionnaires (CPFQ).

The finding that smartphone-based patient-evaluated cognitive difficulties were associated with more perceived stress, lower quality of life and functional capacity in patients with BD was novel. Patients with BD often do not recover full functional capacity despite the absence of affective symptoms, leading to heavy costs due to lost productivity (Wyatt and Henter 1995). Several previous studies have reported on clinical and demographic factors’ impact on work functioning in these patients (Sanchez-Moreno et al. 2009; Elinson et al. 2007; Faurholt-Jepsen et al. 2019). Further, patients experience decreased quality of life despite being in remission, and functional recovery and quality of life have been suggested as important treatment targets for patients with BD (Tse et al. 2014; Depp et al. 2012). The findings from the present study are in line with previous studies suggesting that cognitive impairments are related to poor quality of life in patients with BD (Bonnin et al. 2010; Martinez-Aran et al. 2007; Torrent et al. 2012). This implies that when patients present with subjective cognitive complaints at a clinic visit, it should lead to assessment, evaluation and special attention to stress, quality and function in order to specifically address these important impairments of the patients’ everyday life.

The lack of association between smartphone-based patient-evaluated cognitive function and objectively-measured cognitive performance on neuropsychological tests add to the growing evidence for no or poor association between objective cognitive function and patient-evaluated cognitive function with studies presenting disparity in findings (Demant et al. 2015; Martinez-Aran et al. 2005; Svendsen et al. 2012; van der Werf-Eldering et al. 2011; Burdick et al. 2005; Arts et al. 2011). This points to the need to screen for both subjective and objective cognitive impairments in patients with BD allowing for continuous tracking of changes in cognitive function and response to treatment and/or affective episodes. Also, it points to the need to examine both subjective and objective measures of cognitive functioning in future trials investigating the effect of potential treatment interventions (Miskowiak et al. 2018; 2017).

Using smartphones for this type of monitoring enable clinicians to track cognitive function in detail and real-time with multiple evaluations. Also, it may provide insights into daily variations during prolonged time periods outside the clinical settings allowing clinicians and patients to investigate treatment response and response to affective episodes. However, the brief screening tools used in the present study cannot replace full evaluations of cognition, and rapid changes and/or clear deficits should lead to referral for further thorough assessments including evaluation of other secondary causes of cognitive deficits, as suggested by the International Society of Bipolar Disorders Targeting Cognition Task Force (Miskowiak et al. 2018).

Overall, the present study stresses the importance of considering subjective cognitive problems as relevant for intervention decisions and focusing on stress, quality of life and psychosocial functioning even when no objective impairments are detectable. This seems to be important even when no affective symptoms are present as these are not necessarily the reason for subjective cognitive problems.

### Advantages

The patients included from the two RCTs in the present study were clinically well characterized and were receiving treatment or had received treatment at the Copenhagen Clinic for Affective Disorders, Denmark. During the studies the majority of patients were in full or partly on remission, and thus the cognitive impairments as well as the increased levels of stress, and decreased quality of life and functioning were not due to affective symptoms. The clinical evaluations were conducted multiple times during follow-up by experienced researchers with a specific knowledge within bipolar disorder. Also, the objectively measured cognitive function, functional capacity, depressive and manic symptoms were conducted rater-blinded, since the researchers did not have access to the smartphone data. Further adding to increase the internal validity of the finding of no association between daily patient-evaluated cognitive function measured using smartphones and objectively-measured cognitive function measured using the SCIP, similarly no association was found between subjective measure of cognition using the CPFQ and the observer-based SCIP.

The smartphone-based system used in the present studies (the Monsenso system) were developed by the authors and has been shown easy to use with a high usability, usefulness, ease of learning to use and interface quality–also when compared with other smartphone-based systems (Bardram et al. 2013; Faurholt-Jepsen et al. 2019). The use of smartphones for real-time fine-grained monitoring reduced the risk of recall bias. Since we were not able to conduct statistical power analyses for the present study, multiple testing was taken into account in the statistical analyses.

Overall, the findings from the present study are found to be generalizable to patients with bipolar disorder not presenting with affective episodes who are willing to use a monitoring tool during prolonged time periods.

### Limitations

First, the patients included in the study were recruited as part of two RCTs (the MONARCA I and II trials) investigating the effect of daily smartphone-based monitoring on the severity of depressive and manic symptoms (Faurholt-Jepsen et al. 2019; 2015). Thus, the sample size and follow up period were defined according to the RCTs’ design. It is possible that a larger sample with a longer follow up period could have resulted in other findings. Data on perceived stress and quality of life was collected using questionnaires and due to the nature of the intervention the patients were not blinded to smartphone data. In contrast, clinical assessments of cognitive function, functioning and severity of depressive and manic symptoms were conducted by experienced researchers who were blinded to smartphone data. Second, the smartphone-based scale for monitoring of cognitive function ranged from 0 to 2, limiting the opportunity to investigate associations using a scale with higher granularity. It may be that minor cognitive impairments could have been both over and underestimated by the patients. Along this line, aside from the SCIP we did not include other more comprehensive objective measures of cognitive function. In addition, the patients conducted smartphone-based self-monitoring with an adherence of 66.4%, and it may be that patient-evaluated cognitive function during more or less severe affective states were not collected. However, this is the first study investigating daily parameters in this population. Third, the patients were participating in two RCTs as part of the intervention group and the intervention in itself could have influenced the patients’ symptoms. Fourth, the results are based on association analyses and therefore the cause and consequence between cognitive impairments on the one hand and perceived stress, quality of life and functioning on the other is not entirely clear form the present study. Whether cognitive impairments per se cause increased stress, reduced quality of life and functioning is not clear but could be interesting to investing further in future studies. Fifth, in the statistical analyses the included covariates did not affect the estimates. It may be that other confounding variables could have altered the associations.

### Perspectives

The rapid evolution of smartphone-based technology and the ubiquity of mobile networks have fostered increasing growth of e-mental health technologies including electronic platforms offering tools for remote self-monitoring in BD. Smartphones allow for long-term fine-grained real-time assessments outside the clinical settings (Ebner-Priemer and Trull 2009) and provide unique opportunities to a better understanding of the nature, correlates and clinical implications of cognitive dysfunction in patient with BD. This will add to the opportunity to intervene and potentially initiate treatment to prevent impaired functioning and quality of life in these patients.

## Conclusions

Smartphone-based patients-evaluated cognitive difficulties were associated with more perceived stress, lower quality of life and poorer functional capacity even during full or partly remission. In contrast, there was no association between daily subjective cognitive function measures using smartphones and objective cognitive function in patients with BD. In patients presenting with subjective cognitive complaints evaluation and special attention to stress, quality and functioning should be a priority. Subjective cognitive impairment was associated with clinical disability pointing toward smartphones as a valid tool.

## Data Availability

Not applicable.
